# Enhancing emotion recognition in virtual reality: a multimodal dataset and a temporal emotion detector

**DOI:** 10.3389/fpsyg.2025.1709943

**Published:** 2025-11-24

**Authors:** Chenxin Qu, Xiaoping Che, Yafei Yang, Zhongwei Zhang, Enyao Chang, Jianing Zhang, Hongwei Zhu, Ling Yang

**Affiliations:** 1 Beijing Jiaotong University, Beijing, China; 2 Peking Union Medical College Hospital, Beijing, China; 3 China National Software & Service CO., LTD., Beijing, China

**Keywords:** virtual reality, physiological signals, emotion recognition, deep learning, multimodal data

## Abstract

Emotion is a complex psychophysiological phenomenon elicited by external stimuli, exerting a profound influence on cognitive processes, decision-making, and social behavior. Emotion recognition holds broad application potential in healthcare, education, and entertainment. With virtual reality (VR) emerging as a powerful tool, it offers an immersive and controllable experimental environment. Prior studies have confirmed the feasibility and advantages of VR for emotion elicitation and recognition, and multimodal fusion has become a key strategy for enhancing recognition accuracy. However, publicly available VR multimodal emotion datasets remain limited in both scale and diversity due to the scarcity of VR content and the complexity of data collection. The shortage hampers further progress. Moreover, existing multimodal approaches still face challenges such as noise interference, large inter-individual variability, and insufficient model generalization. Achieving robust and accurate physiological signal processing and emotion modeling in VR environments thus remains an open challenge. To address the issues, we constructed a VR experimental environment and selected 10 emotion-eliciting video clips guided by the PAD(Pleasure-Arousal-Dominance) model. Thirty-eight participants (N=38) were recruited, from whom electrodermal activity, eye-tracking, and questionnaire data were collected, yielding 366 valid trials. The newly collected dataset substantially extends the publicly available VREED dataset, enriching VR-based multimodal emotion resources. Furthermore, we propose the MMTED model (Multi-Modal Temporal Emotion Detector), which incorporates baseline calibration and multimodal fusion of electrodermal and eye-tracking signals for emotion recognition. Experimental results demonstrate the strong performance of the MMTED model, achieving accuracies of 85.52% on the public VREED dataset, 89.27% on our self-collected dataset, and 85.29% on their combination.

## 1 Introduction

Emotion is a complex psychophysiological phenomenon elicited by external stimuli, encompassing subjective experiences, behavioral expressions, and physiological alterations (Shu et al., 2018). As a fundamental psychological state, it profoundly influences individual cognition, decision-making, and social interactions (Lerner et al., 2014). Therefore, advancing emotion recognition technologies holds great significance for understanding human affective mechanisms and optimizing human-computer interaction. Such technologies have demonstrated broad application potential in diverse fields, including mental health assessment and psychotherapy (Praveena et al., 2020), education and adaptive learning (Arduini and De Vito, 2024), and affect-aware human-computer interaction(Yang et al., 2023). Virtual reality (VR) has emerged as a powerful tool due to its immersive, interactive, and controllable environments. Unlike traditional two-dimensional media stimuli (Uhrig et al., 2016; Romeo et al., 2022), VR engages multiple sensory channels, including visual, auditory, and even haptic modalities, to evoke more natural and stable affective states. Empirical studies have shown that VR can effectively elicit both basic emotions (e.g., fear, joy) and complex affective states (e.g., tension, excitement) (Rivu et al., 2021; Somarathna et al., 2022). Moreover, VR often produces stronger physiological and self-reported responses compared to traditional media, particularly in the induction of high-arousal negative emotions (Magdin et al., 2021). The advantages make VR a reliable experimental paradigm for emotion elicitation and recognition research, offering enhanced ecological validity and experimental control.

To achieve accurate recognition of emotional states, researchers have explored three major methodological approaches: subjective evaluation, behavioral analysis, and physiological signal analysis. Subjective evaluation methods collect self-reported emotional states through questionnaires or rating scales, such as the Self-Rating Emotional Scale (Gross and John, 2003) and the Positive and Negative Affect Schedule (PANAS) (Partala and Kallinen, 2012). Behavioral analysis infers emotions from observable features such as facial expressions, gestures, or vocal characteristics. Physiological signal analysis, by contrast, examines changes in biosignals including skin conductance, heart rate variability, and electroencephalogram (EEG) activity (Alarcao and Fonseca, 2017; Zhao et al., 2016). Within VR environments, the approaches face unique opportunities and challenges. Subjective evaluation methods remain effective in VR settings, where users typically complete pre- and post-experience questionnaires to provide feedback. However, behavioral analysis encounters certain constraints in VR: head-mounted displays (HMDs) partially obscure the upper face, which limits the effectiveness of traditional facial expression recognition (Ortmann et al., 2025). Researchers explored indirect emotion recognition approaches such as eye movement data, vocal characteristics, and head motion. In contrast, physiological signal analysis demonstrates high adaptability in VR. Signals such as electrocardiogram (ECG), electrodermal activity (EDA), and electroencephalogram (EEG) can be captured without disrupting the immersive experience. Therefore, physiological signal analysis becomes the predominant method in current VR-based emotion recognition research (Gao et al., 2020; Halbig and Latoschik, 2021). In practice, the emotional data can serve as objective feedback for the design of VR content, enabling developers to identify scenarios that elicit positive or negative emotional responses. Furthermore, it can facilitate the development of targeted content for emotionally adaptive virtual environments.

Immersive VR environments further enable the integration of physiological and behavioral data, providing rich multimodal information for emotion recognition. Recent studies leveraging multimodal signals with deep learning architectures have reported promising results in VR settings (Marín-Morales et al., 2020; Polo et al., 2025; Li et al., 2025). However, despite the advances, the field faces persistent bottlenecks due to the scarcity of large-scale, high-quality VR emotion datasets. Existing datasets, such as VREED (Tabbaa et al., 2021), DER-VREEG (Suhaimi et al., 2022), and VRMN-bD (Zhang et al., 2024), are constrained by limited modalities, small participant pools, or narrow emotional categories. As a result, they remain insufficient to support the development of robust and generalizable recognition models (Gnacek et al., 2024). Although unimodal and multimodal methods have shown potential (Marín-Morales et al., 2021; Geraets et al., 2021; Wang and Wang, 2025), challenges such as class imbalance, and individual variability hinder progress. The paper seeks to address the two major issues by contributing new data collection and proposing a novel fusion-based framework.

To complement existing multimodal emotion datasets in VR environments, the study was designed with reference to VREED. A pre-experiment was conducted to select 10 emotion-eliciting clips from 20 VR videos. 38 volunteers were recruited to watch the clips while wearing VR headsets. During the experiment, participants' real-time galvanic skin response (GSR) and eye-tracking data were recorded, and subjective feedback was additionally collected through questionnaires. In total, 366 valid samples were obtained. Furthermore, we developed a multimodal emotion recognition network. The model first extracts features from eye-tracking and GSR data, and then integrates them through a multimodal feature fusion strategy to achieve efficient emotion representation. The effectiveness of the proposed method was validated on the VREED dataset, the self-constructed dataset, and their combination. In summary, the paper makes two main contributions:

- We construct a new multimodal VR emotion dataset by selecting validated VR video stimuli, yielding 366 valid samples to supplement existing datasets VREED.
- We propose a multimodal emotion recognition framework, MMTED, which integrates features from GSR and eye-tracking data via feature fusion strategies for accurate emotion.

## 2 Related works

### 2.1 Emotion Recognition in virtual environments

In recent years, emotion recognition in virtual reality has garnered significant scholarly attention. Related studies utilize VR technology to construct interactive environments, designing various scenarios controlled experimental conditions to elicit, measure, and analyze users' emotional responses (Somarathna et al., 2022).

Currently, numerous studies are focused on developing emotion-inducing tasks within virtual environments and validating their effectiveness through a combination of subjective assessments and physiological signal analysis. For example, (Liao et al., 2019) developed a VR environment with targeted emotional induction and validated its ecological validity advantage through multimodal physiological measures and self-report questionnaires. Their experiment found that the VR environment was more effective in evoking target emotional responses compared to traditional video stimuli. (Felnhofer et al., 2015) manipulated environmental variables such as lighting, weather, and time of day in a virtual park to induce five emotions: joy, sadness, boredom, anger, and anxiety. They confirmed the effectiveness of the environments in eliciting multiple emotional states and explored the relationship between physiological indicators, such as skin conductance, and emotional arousal and presence. (Dozio et al., 2022) proposed a method for constructing emotional virtual environments by integrating emotional theory and semantic elements. They validated its effectiveness across the valence, arousal, and dominance dimensions. Their work emphasized the development of ten specific emotion-inducing scenes using primarily visual and auditory stimuli to facilitate user emotion elicitation. (Marcolin et al., 2021) highlighted the importance of emotional dimensions in affective VR design, noting that different emotions require distinct design strategies. For instance, fear-inducing scenes often incorporate dark color schemes and sudden stimuli, whereas joyful environments utilize bright colors and engaging elements. They conducted experiments in both immersive and non-immersive VR settings, collecting ECG, EDA, and EEG signals along with subjective ratings to validate their application. (Lima et al., 2024) used 52 silent video clips from the EMDB database, covering positive, negative, and neutral emotions such as happiness, sadness, fear, anger, and disgust. The stimuli were presented in both immersive and non-immersive VR environments, with participants' physiological signals collected and analyzed for emotion recognition.

Some studies also concentrate on constructing virtual emotion databases, aiming to establish a standardized foundation for emotion annotation to support subsequent algorithm training. (Suhaimi et al., 2018) constructed a VR-based emotion recognition database using the arousal-valence model to annotate participants' emotional states, thereby providing critical support for the development of VR-based emotion classification algorithms. (Radiah et al., 2023) developed a Unity3D-based system containing six distinct emotional scenarios, covering typical emotional states from happiness to fear. They employed the Self-Assessment Manikin (SAM) scale to evaluate emotional intensity. (Tabbaa et al., 2021) publicly released the VREED dataset, which captures the emotional responses of 34 volunteers in an immersive 360° video virtual environment (360-VES) by recording 59-channel GSR and ECG data. Currently, the dataset is one of the few multi-emotion databases available in the field of virtual reality research.

Overall, existing research has preliminarily confirmed the feasibility and advantages of virtual reality in emotion induction and recognition. By constructing immersive virtual scenarios, researchers can effectively elicit specific emotional responses in controlled experimental settings. The findings have laid a solid foundation for emotion recognition based on virtual environments. However, certain limitations remain. For instance, some studies employ limited or repetitive emotional scenarios, a scarcity of VR-based emotional datasets with limited sample sizes, the use of homogeneous emotion induction scenarios, and a heavy reliance on traditional metrics and self-report questionnaires. Moreover, many approaches have yet to fully leverage the capability of deep learning in mining and integrating multimodal physiological signals.

### 2.2 Physiological signal-based emotion recognition

Compared to traditional methods based on facial expressions and vocal signals, physiological signals offer advantages such as objectivity, stability, and resistance to disguise, thereby improving the accuracy and ecological validity of emotion recognition.

Existing studies have explored the application of various physiological indicators in virtual environments. Among these, galvanic skin response (GSR) is an important signal reflecting autonomic nervous system activity and indicative of emotional arousal levels. (Hinkle et al., 2019) demonstrated that GSR is closely linked to emotional arousal and can effectively differentiate between emotions. In their VR-based experiments, support vector machines and k-nearest neighbors algorithms are used to classify GSR signals, they achieved a maximum recognition accuracy of 89.19Eye movement data, as a non-invasive physiological behavioral indicator, also demonstrates strong potential for emotion recognition. (Zheng et al., 2020) applied eye-tracking technology to detect users' emotional states and found that features such as pupil dilation and eye movement trajectory correlate with emotional valence. Positive emotions are typically associated with pupil dilation, whereas negative emotions tend to result in pupil constriction. The eye movement characteristics provide valuable insights for real-time assessment of users' emotional conditions. Electroencephalogram (EEG) signals possess inherent advantages in capturing emotional and cognitive processes. (ZHAO Dan-Dan, 2023) segmented the frequency bands of preprocessed EEG signals, extracted differential entropy features, and developed a hybrid model combining convolutional neural networks and gated recurrent units to classify emotions into three categories: positive, negative, and neutral. The model achieved an average recognition accuracy of 86.50% on the SEED dataset, though its cross-subject generalization capability remains limited.

Consequently, multimodal fusion approaches have gradually emerged as a key strategy for improving emotion recognition performance. (Cimtay et al., 2020) proposed a hybrid model integrating multimodal signals including facial expressions, GSR, and EEG. Applied to the DEAP dataset, their model achieved a maximum accuracy of 91.5% in classifying emotions such as anger, disgust, fear, happiness, neutrality, sadness, and surprise, surpassing the performance of single-modal models. The highlights the advantage of multimodal fusion in complex emotion recognition tasks. Similarly, (Arslan et al., 2024) combined ECG and GSR signals to recognize five emotional states—excitement, happiness, anxiety, calmness, and sadness—in a VR environment. Although overall accuracy was high, the model struggled with blurred boundaries and exhibited limited generalization capability.

Despite the strong application prospects of physiological signal-based emotion recognition in VR contexts, it still faces significant challenges such as inherent signal noise, pronounced inter-subject variability, and consequently limited model generalization. The acquisition of physiological data, including GSR and eye-tracking signals, is highly susceptible to contamination from motion artifacts induced by user interaction. Furthermore, the high degree of individual differences in physiological responses makes it difficult to build a universal model, often leading to biased performance when applied to new users. Achieving robust and high-precision physiological signal processing and emotion modeling in virtual environments remains a critical issue to be addressed.

### 2.3 Deep learning-based emotion recognition

With VR advancements, emotion recognition research is expanding into more complex and realistic interactive environments. Deep learning methods, leveraging strengths in multimodal data modeling and temporal feature extraction, have become vital for precise emotion recognition in VR.

In deep learning, convolutional neural networks (CNNs) and long short-term memory (LSTM) networks excel in processing temporal signals and multimodal data. CNNs perform well in time-frequency feature extraction. For instance, (Dessai and Virani, 2023) improved emotion classification accuracy by converting GSR signals to time-frequency domains via continuous wavelet transform and applying CNNs. (Tang et al., 2017) demonstrated LSTM's effectiveness in modeling physiological signal dynamics to capture emotional temporal dependencies. (Li, 2024) combined CNN and LSTM into a CRNN model using fast Fourier transform features, achieving higher accuracy than standalone CNN or LSTM models.

Recent trends emphasize multimodal fusion. (Zhang et al., 2020) transformed GSR and eye movement data into network-compatible inputs, using deep models to learn latent features and improve complex emotion discrimination. (Zali-Vargahan et al., 2023) extended modalities by fusing EEG, peripheral physiological signals, and facial expressions, converting signals to time-frequency images via 2D discrete Stockwell transform and extracting deep features via CNN, achieving 95.3% and 92.8% accuracy for binary and four-class classification on DEAP. (Ma et al., 2019) proposed a multimodal residual LSTM network with shared weights to learn EEG-physiological signal correlations, achieving 92.87% and 92.30% accuracy for arousal and valence classification on DEAP, outperforming traditional LSTM.

While deep learning excels in multimodal feature extraction and fusion, current research primarily focuses on coarse-grained emotion classification, with limited capability for fine-grained, multi-category emotion recognition.

## 3 Dataset collection

### 3.1 Experiment design

To accurately characterize emotional states, the PAD (Pleasure-Arousal-Dominance) model was employed. The model posits that emotions can be described along three continuous dimensions: pleasure, arousal, and dominance. The Pleasure dimension, also referred to as valence, indicates the positivity or negativity of an emotional state, ranging from one extreme (e.g., distress) to the other (e.g., ecstasy). The Arousal dimension, alternatively termed activation, captures the level of physiological and psychological alertness. States such as sleep and boredom are associated with low arousal, while wakefulness and tension correspond to high arousal. The dominance dimension, sometimes labeled as the control or influence dimension, indicates the extent to which an individual feels in control of or controlled by their environment or others. High dominance is associated with feelings of empowerment, such as anger and bravery, whereas low dominance is linked to states of vulnerability, such as anxiety and fear.

**Figure 1:**
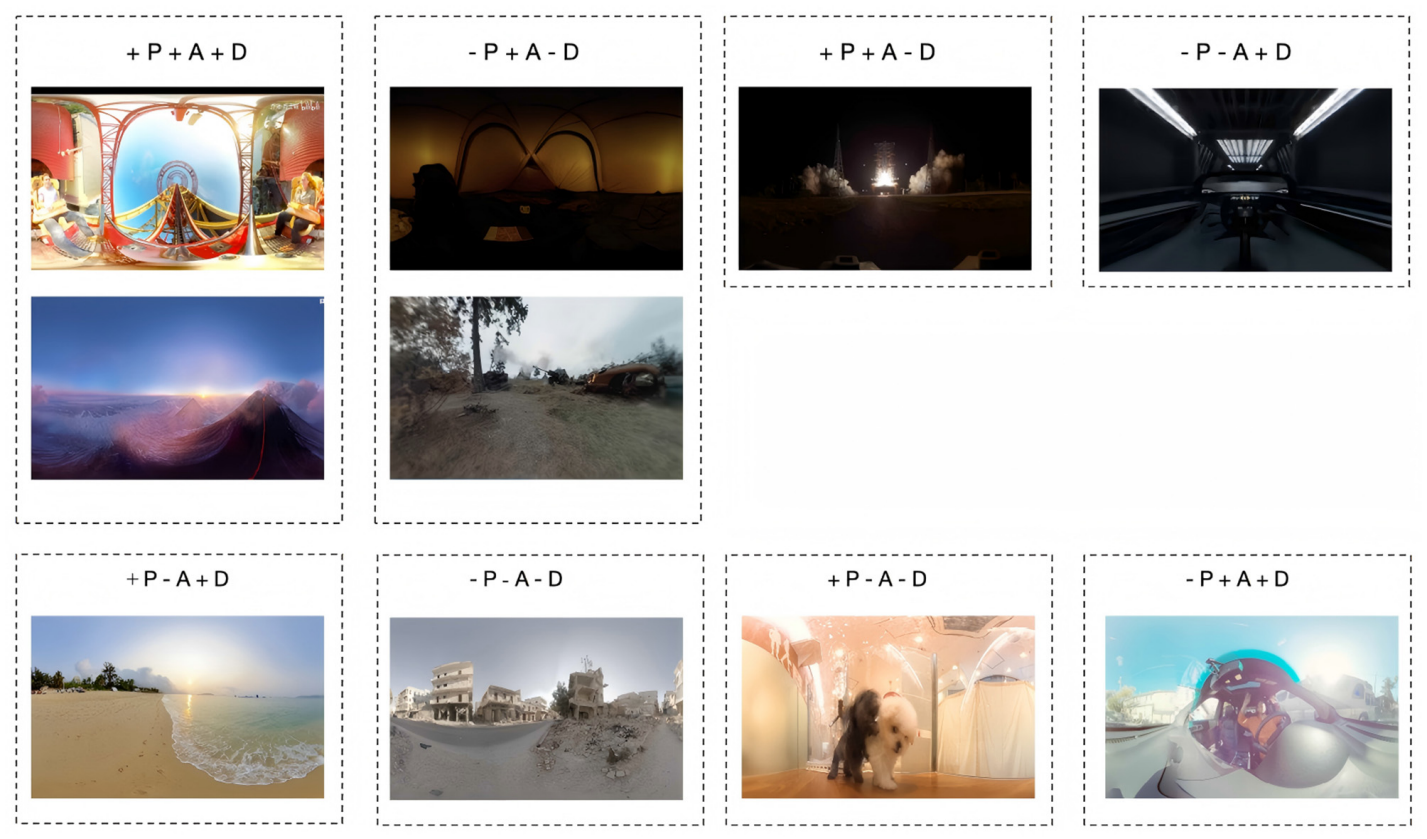
Selected videos for the formal experiment.

20 VR videos with distinct emotional tendencies were carefully selected from online platforms and public databases, covering a wide range of emotional states, from high pleasure, high arousal, and high dominance to low pleasure, low arousal, and low dominance.^1^,^2^ The design of situational elements such as lighting, tone, perspective, and rhythm in the videos was fully informed by established guidelines from prior research on emotion-inducing environments (Romeo et al., 2022; Rivu et al., 2021). Prior to the formal experiment, a pilot study was conducted to evaluate and select suitable experimental videos. A total of ten volunteers participated in the preliminary phase. Each participant wore a VR headset to experience various video types and completed an emotional self-assessment questionnaire immediately after each viewing. The data collected during the pilot study were used to assess the emotional elicitation effectiveness of each video. The selection process was conducted subjectively, based on whether the scores across the three dimensions (P, A and D) of the 9-Likert-scale self-assessment questionnaire showed significant differentiation, as well as on the technical stability and immersive quality of the videos themselves. Based on the criteria, 10 video clips (in Figure 1) demonstrating superior emotional elicitation validity and visual immersion were selected from the original pool of 20 candidates to constitute the final experimental stimulus set, as detailed in Table 1. Videos with identifiers beginning with “V” denote the final selected materials. The video library covers all eight possible combinations of the three dimensions of P, A, and D, including their positive and negative variations ±P, ±A, ±D.

**Table 1:** Library of VR emotion-eliciting videos.

**ID**	**Name**	**PAD**	**P**	**A**	**D**
V01	The Soviet-German battlefield in World War II	−P +A −D	2.4 ± 0.6	7.6 ± 0.5	3.1 ± 0.6
V02	Concept car experience	−P −A +D	3.2 ± 0.5	2.9 ± 0.4	7.1 ± 0.5
V03	Roller coaster experience	+P +A ±D	6.8 ± 0.7	8.2 ± 0.4	5.6 ± 0.8
V04	eruption	+P +A +D	7.0 ± 0.5	7.9 ± 0.6	6.9 ± 0.5
V05	escape from prison	−P +A +D	3.5 ± 0.7	7.1 ± 0.6	6.2 ± 0.6
V06	Interact with a puppy	+P −A −D	8.2 ± 0.5	3.5 ± 0.5	4.0 ± 0.6
V07	forest	−P +A −D	2.7 ± 0.6	7.3 ± 0.4	3.3 ± 0.6
V08	Post war ruins	−P −A −D	2.1 ± 0.5	3.1 ± 0.6	2.5 ± 0.6
V09	beach	+P −A +D	7.5 ± 0.5	3.1 ± 0.6	6.7 ± 0.4
V10	rocket launching	+P +A −D	6.5 ± 0.4	8.2 ± 0.4	4.2 ± 0.5

### 3.2 Questionnaire design

The paper employed structured questionnaires to quantify the subjective experiences and individual characteristic variables of participants throughout the experimental process. The questionnaire system consisted of four distinct instruments: one pre-test questionnaire and three post-test questionnaires. The pre-test questionnaire was designed to be completed before the formal experiment and aimed to collect base information of participants. The post-test questionnaires were completed after viewing each experimental video, and were used to evaluate subjective emotional states, sense of presence, and emotional responses within the virtual reality environment.

The pre-test questionnaire consisted of a total of 12 items (see Table 2), including basic personal information, familiarity with VR and the video stimuli, and individual immersion propensity. Specifically, the immersion tendency section was adapted from the Immersion Tendency Questionnaire (ITQ) scale (Russell, 1980), and a five-point Likert scale was employed to quantify subjective traits. To control for potential confounding effects of familiarity with video content on emotional responses, an item measuring “familiarity with the video content to be viewed” was included in the pre-test and used as a covariate in subsequent analyses.

**Table 2:** Pre-test User Questionnaire

**ID**	**Item**
1	Field of study/professional background.
2	Do you frequently use VR devices or experience VR content?
3	I tend to become easily absorbed in the plot of movies or TV shows.
4	I tend to be less affected by external distractions when immersed in an activity.
5	I often lose track of time during immersive experiences.
6	In states of immersion, my emotions fluctuate noticeably in response to environmental stimuli.
7	I typically experience heightened realism or a sense of “being there” when viewing VR content.
8	I believe the level of immersion influences my emotional responses to content.
9	I am familiar with the content of the VR videos I am about to watch.

**Figure 2:**
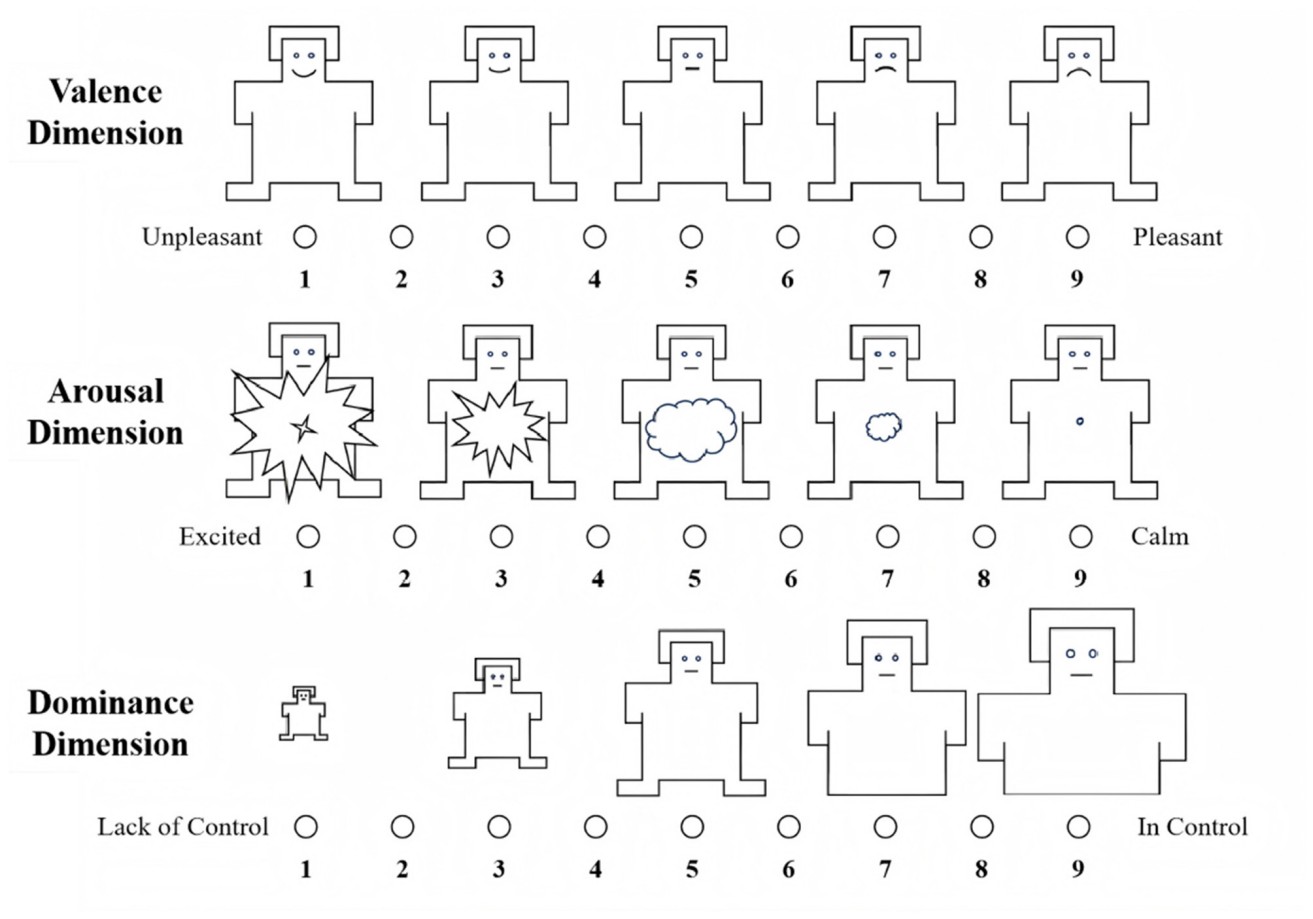
SAM scale.

The post-test questionnaires were used to assess participants' emotional changes and experiential feedback after watching each VR video. After watching each video, participants were required to complete three separate questionnaires:

- The Self-Assessment Manikin (SAM) Scale, which uses pictorial representations to measure emotional responses along the dimensions of pleasure, arousal, and dominance (see Figure 2).
- The PAD (Pleasure-Arousal-Dominance) Scale, designed to capture the direction and intensity of the participants' subjective emotions during viewing (see Table 3).
- The Presence Questionnaire, which included items such as “sense of being there,” “reduced awareness of reality,” and “environmental realism” (see Table 3).

**Table 3:** PAD emotion scale.

**ID**	**Emotion**	**-4**	**-3**	**-2**	**-1**	**0**	**1**	**2**	**3**	**4**	**Emotion**
1	Angry	°	°	°	°	°	°	°	°	°	Activated
2	Wide	°	°	°	°	°	°	°	°	°	Sleepy
3	Controlled	°	°	°	°	°	°	°	°	°	Controlling
4	Friendly	°	°	°	°	°	°	°	°	°	Scornful
5	Calm	°	°	°	°	°	°	°	°	°	Excited
6	Dominant	°	°	°	°	°	°	°	°	°	Submissive
7	Cruel	°	°	°	°	°	°	°	°	°	Joyful
8	Interested	°	°	°	°	°	°	°	°	°	Relaxed
9	Guided	°	°	°	°	°	°	°	°	°	Autonomous
10	Excited	°	°	°	°	°	°	°	°	°	Enraged
11	Relaxed	°	°	°	°	°	°	°	°	°	Hopeful
12	Influential	°	°	°	°	°	°	°	°	°	Influenced

Based on the collected questionnaire data, emotional labels were quantitatively processed. A total of 12 items (Q1-Q12) from the PAD-based questionnaire were used to characterize the three emotional dimensions. The values for each dimension were calculated according to the Equations 1 –3.

Pleasure:


$$\begin{array}{l} P=\frac{Q_{1}-Q_{4}+Q_{7}-Q_{10}}{4} \end{array}$$ (1)

Arousal:


$$\begin{array}{l} A=\frac{-Q_{2}+Q_{5}-Q_{8}+Q_{11}}{4} \end{array}$$ (2)

Dominance:


$$\begin{array}{l} D=\frac{Q_{3}-Q_{6}+Q_{9}-Q_{12}}{4} \end{array}$$ (3)

To support model training in classification tasks, the study maps PAD coordinates to discrete emotion categories by calculating the Euclidean distances between individual PAD coordinates and predefined emotion prototype points. Each PAD coordinate is assigned the label of the emotion category whose prototype is closest in the emotional space. The mapping process effectively converts subjective assessment results into categorical labels while preserving the continuity of emotional expression, thereby establishing an emotion labeling framework suitable for machine learning. The approach facilitates the subsequent training and evaluation of emotion recognition models.

### 3.3 Ethical review

Given the strong socio-humanistic dimensions of the experiment, which involves participants' psychological well-being and privacy protection, the experimental procedures were strictly standardized to safeguard the safety of the subjects and the credibility of the results. The study implemented ethical review and professional evaluations before the experiment.

After clarifying the experimental process, the research team invited experts from the Department of Psychology and the field of cognitive neuroscience at Peking Union Medical College Hospital, Chinese Academy of Medical Sciences, to evaluate the experimental design and materials. Particular attention was given to assessing the potential psychological effects of the video content on participants. Upon expert review, it was confirmed that none of the materials contained violent, frightening, or trauma-inducing scenes, thereby ensuring that the experiment would not induce long-term adverse psychological effects.

To protect participant privacy, all data collected throughout the study were anonymized and subjected to strict access control measures.

### 3.4 Experiment procedure

The study recruited a total of 38 participants through online social platforms and campus bulletins. All volunteers had normal auditory and visual abilities, with no history of severe visual impairment or neurological disorders. Prior to the experiment, the research staff once again explained the purpose and procedures of the study to the participants. Each volunteer was required to sign an Informed Consent Form, which detailed the specific content of the experiment, expected duration, potential risks, participant rights, and the scope of data usage. Participants were explicitly informed of their right to terminate the experiment at any time. All collected data were anonymized, stored solely for research purposes, and not used for any commercial applications.

**Figure 3:**
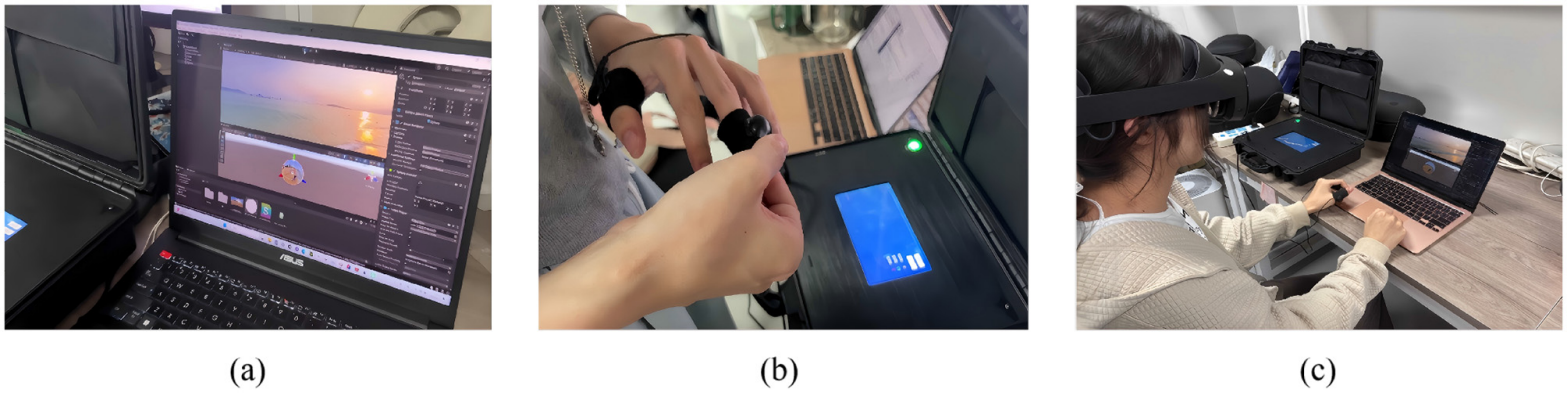
Experiment procedure. **(a)** Multi-dimensional emotion elicitation scenario, **(b)** GSR sensor, **(c)** Participants in the experiment.

The study employed the Meta Quest Pro as the primary head-mounted display (HMD) and utilized the Unity platform to develop multi-dimensional emotion elicitation scenarios (see in Figure 3a). By integrating functionalities such as video playback and interactive logic within a unified engine, the experimental procedure achieved both stability and flexibility, while also facilitating future scalability. For physiological data acquisition, a jskj-pf GSR sensor was used to capture electrodermal activity in real time. The sensor was attached to participants' fingers to continuously monitor changes in skin conductance under various emotional stimuli(see in Figure 3b). Eye movement data were collected using a plugin integrated within the Unity environment. Metrics such as gaze position, fixation duration, pupil diameter variation, and blink frequency were recorded. The parameters provide insights into the distribution of attention and psychological responses in specific emotional contexts.

The experiment consists of three stages: the pre-test, the video viewing, and the post-test stage. During the pre-test stage, participants read the experimental instructions, signed the informed consent form, became familiar with the physiological signal acquisition equipment and HMD, and completed the pre-test questionnaire. In the video viewing stage, each participant was required to watch 10 videos. The viewing order was randomized for each participant. After being fitted with the GSR sensor, participants viewed the videos via a VR headset (see in Figure 3c). During the time, real-time EDA and eye movement signals were recorded. In the post-test stage, immediately after each video was played, participants completed three subjective questionnaires: the Self-Assessment Manikin (SAM), the Positive and Negative Affect Schedule (PAD), and a presence questionnaire. They then proceeded to the next video and subsequent questionnaire round. To mitigate fatigue effects, a short rest interval was provided after each video. The total duration of the experiment for each participant was maintained within 60 minutes.

### 3.5 Data preprocessing

As an essential physiological indicator reflecting the arousal state of the autonomic nervous system, the galvanic skin response (GSR) signal is highly sensitive to emotional fluctuations. After baseline correction and standardization, the following features were extracted from the raw signals: Mean GSR value, which reflects the overall level of sympathetic arousal; Standard deviation and variance, indicating the intensity of signal fluctuations and dispersion, representing the magnitude of emotional changes; Minimum and maximum values, capturing the lowest inhibition point and the peak activation point under emotional stimuli. Due to baseline correction, the values may be negative. Number of peaks and troughs, representing the frequency of rapid increases and decreases in GSR during stimulation, which reveals the intensity of physiological reactivity to emotional stimuli; Peak-to-trough ratio, used to measure the dynamic symmetry between rapid excitation and recovery of the GSR signal, reflecting the symmetry and trend of arousal responses. See Equation 4:


$$\begin{array}{l} Ratio=\frac{Number\;of\;Peaks}{Number\;of\;Valleys+\epsilon } \end{array}$$ (4)

where ϵ is a small constant to prevent division by zero.

Each segment of the GSR signal was ultimately transformed into a structured feature vector, affixed with a corresponding emotional category label, and used for subsequent training of machine learning models and emotion recognition tasks.

Eye movement data serve as a key indicator for revealing an individual's cognitive load, attention distribution, and emotional state.

Regarding fixation characteristics, Identification by Dispersion-Threshold (I-DT) was employed to identify fixation segments. A threshold was predefined, and contiguous points where the angular velocity remained below the value for a certain duration were classified as a fixation. Angular velocity was calculated using Equation 5:


$$\begin{array}{l} \omega_{i}=\frac{\sqrt{{\left(x_{i+1}-x_{i}\right)}^{2}+{\left(y_{i+1}-y_{i}\right)}^{2}}}{t_{i+1}-t_{i}} \end{array}$$ (5)

where (*x*_*i*_, *y*_*i*_) and (*x*_*i*+1_, *y*_*i*+1_) denote the coordinates of the user's gaze points at times *t*_*i*_ and *t*_*i*+1_ respectively. For all detected fixation segments, statistical measures were extracted, including the number of fixations, mean fixation duration, standard deviation, skewness, maximum fixation duration, and first fixation duration. The metrics reflect the participant's information uptake density and visual dwelling preferences.

Saccade Characteristics: Non-fixation segments were classified as saccades. A minimum duration threshold was applied, and the following saccade features were extracted: number of saccades, saccade duration, amplitude, direction angle, and path length. Saccade amplitude and direction were calculated based on the angular difference between the starting and ending gaze vectors, while the path length was derived from the cumulative angular changes along the saccade trajectory. The metrics help reveal cognitive strategies during rapid visual information integration. The saccade amplitude was computed using the spherical vector angle formula, as shown in Equation 6:


$$\begin{array}{l} A=arccos\left(\frac{\vec{g_{s}}\cdot \vec{g_{e}}}{\left|\vec{g_{s}}|\cdot |\vec{g_{e}}\right|}\right)\times \frac{180}{\pi } \end{array}$$ (6)

where $\vec{g_{s}}$ and $\vec{g_{e}}$ end denote the unit gaze vectors at the beginning and end of the saccade respectively.

Regarding blink characteristics, since the raw data did not include annotated blink events, an adaptive threshold was established based on the distribution of binocular Z-axis velocity changes to detect abrupt shifts indicative of blinks. The identified blink segments were used to compute metrics such as blink count, mean blink duration, standard deviation, skewness, and maximum duration. Among these, blink frequency and duration can reflect the participant's fatigue level, emotional fluctuations, and changes in alertness.

To enhance the model's ability to capture directional details in eye movements, the angular changes of saccades were computed separately along the horizontal (X-axis) and vertical (Y-axis) directions. The approach helps characterize directional biases in visual scanning, reflecting underlying tendencies in attentional allocation.

Skewness (see Equation 7) and kurtosis (see Equation 8) are used to characterize the asymmetry and outlier distribution of eye movement behavior. The formulas are as follows:


$$\begin{array}{l} Skewness=\frac{\frac{1}{n}\sum {\left(x_{i}-\bar{x}\right)}^{3}}{{\left(\frac{1}{n}\sum {\left(x_{i}-\bar{x}\right)}^{2}\right)}^{3/2}} \end{array}$$ (7)

$$\begin{array}{l} Skewness=\frac{\frac{1}{n}\sum {\left(x_{i}-\bar{x}\right)}^{4}}{{\left(\frac{1}{n}\sum {\left(x_{i}-\bar{x}\right)}^{2}\right)}^{2}} \end{array}$$ (8)

To account for the synergistic or compensatory relationship between head and eye movements in certain scenarios, head rotation components (Head Rotation X/Y/Z/W) derived from quaternion analysis were incorporated into the feature set. The rate of change per unit time was computed as dynamic head motion features, capturing the influence of head movement on gaze behavior. Statistical features were extracted within each time window.

All eye movement features were sampled at the frame level, processed using sliding-window statistics, and subjected to outlier removal, resulting in stable and reliable individual feature sequences. During acquisition, both the GSR sensor and eye-tracker were synced to the VR system clock. We downsampled the eye data to match the GSR sampling rate by grouping gaze samples into 1-second windows aligned with GSR timestamps. Conversely, for each eye frame we associated the nearest GSR value. This ensured that each feature vector in our model had correctly paired eye and GSR inputs.

## 4 Multi-modal temporal emotion detector

**Figure 4:**
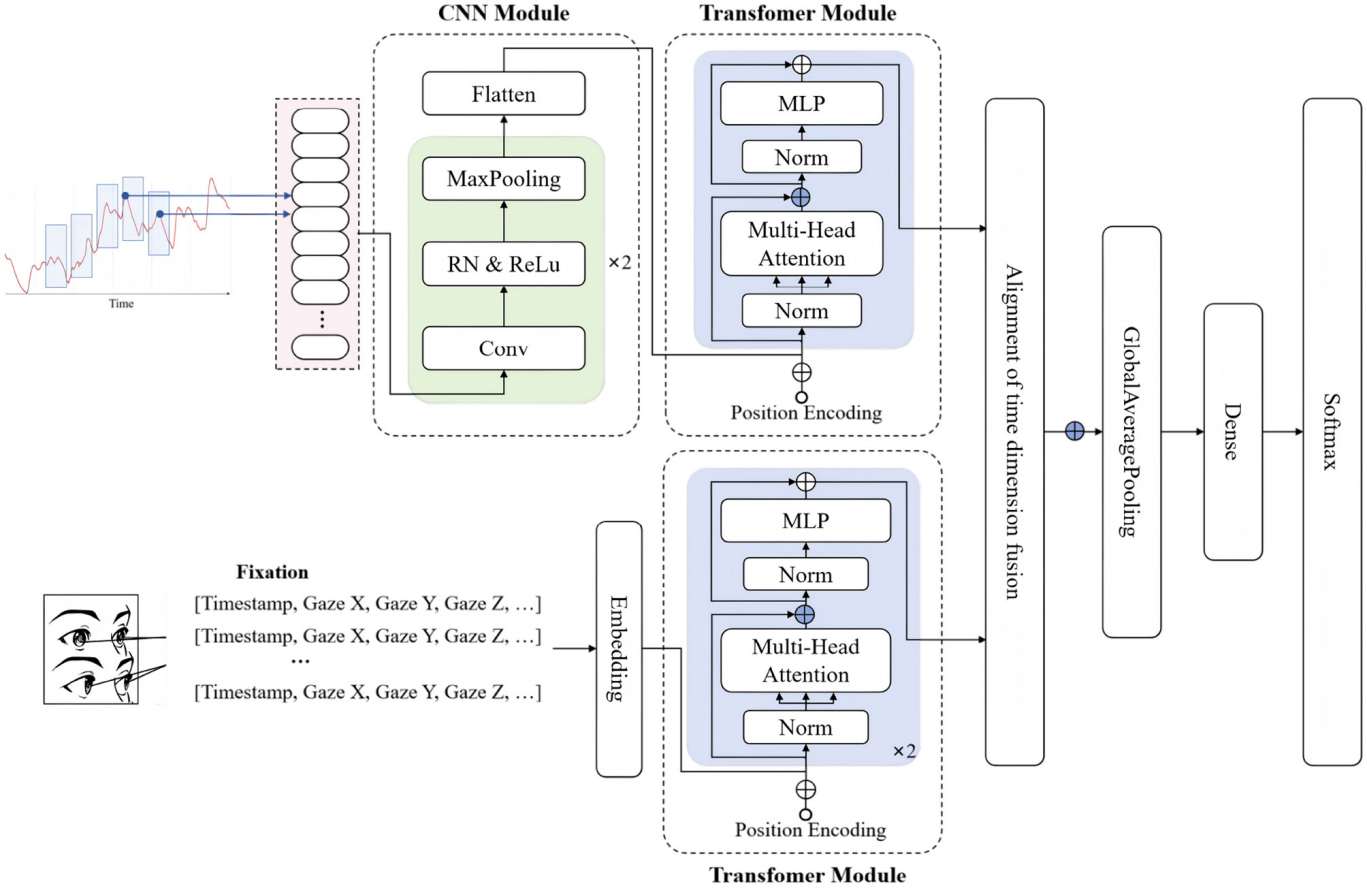
MMTED: multi-modal temporal emotion detector.

To mitigate potential information loss associated with manual feature engineering, the paper adopts a raw time-series-based modeling approach Multi-Modal Temporal Emotion Detector (MMTED). In MMTED, an end-to-end architecture is employed to automatically learn salient temporal features directly from the input signals. The overall model structure consists of three components: GSR feature extraction, eye movement feature extraction, and feature concatenation and output, as illustrated in Figure 4.

The GSR feature extraction module comprises two parts: A convolutional layer that captures local trends and short-term dynamic patterns, and a Transformer module that models global dependencies and temporal evolutionary structures. The standardized GSR signal sequences are fed into a one-dimensional convolutional layer containing multiple kernels and ReLU activation functions. The module primarily extracts key morphological characteristics such as local fluctuations and variation rates, as specified in Equations 9, 10.


$$\begin{array}{l} H_{1}=ReLU\left(Conv1D_{1}\left(X_{in}\right)\right) \end{array}$$ (9)

$$\begin{array}{l} H_{2}=ReLU\left(Conv1D_{1}\left(H_{1}\right)\right) \end{array}$$ (10)

Where the receptive field of the first convolutional kernel is set to k = 5, and that of the second kernel to k = 3. The first layer contains 64 convolutional kernels, while the second layer contains 128. The output feature sequence from the convolutional layer, denoted as *H*_2_, has the shape *R*^*B*×*T*×*d*^, where d represents the output dimension of the convolution.

The output is then fed into a Transformer module, which integrates a multi-head self-attention mechanism with feed-forward networks. The structure enables the model to capture long-range dependencies between different time steps in the sequence and dynamically adjust the focus on critical segments based on self-attention scores. Given an input H, the attention output is given by Equation 11:


$$\begin{array}{l} Attention\left(Q,K,V\right)=softmax\left(\frac{QK^{T}}{\sqrt{d_{k}}}\right)V \end{array}$$ (11)

where:


$$\begin{array}{l} Q=HW^{Q},K=HW^{K},V=HW^{V} \end{array}$$

Then the multiple attention heads are processed in parallel and subsequently concatenated, as expressed in Equation 12.


$$\begin{array}{l} MultiHead\left(H\right)=Concat\left(head_{1},\ldots ,head_{h}\right)W^{0} \end{array}$$ (12)

Each encoder layer's output is connected with residual connections and layer normalization., as shown in Equations 13, 14:


$$\begin{array}{l} H^{\prime }=LayerNorm\left(H+MultiHead\left(H\right)\right) \end{array}$$ (13)

$$\begin{array}{l} H^{\prime \prime }=LayerNorm\left(H+FFN\left(H^{\prime }\right)\right) \end{array}$$ (14)

where FFN denotes the feedforward neural network:


$$\begin{array}{l} FFN\left(x\right)=max\left(0,xW_{1}+b_{1}\right)W_{2}+b_{2} \end{array}$$ (15)

The model employs a 2-layer stacked Transformer encoder with 4 attention heads and a feed-forward dimension of 256. The temporal representation produced by the Transformer is condensed into a fixed-length representation via global average pooling, as specified in Equation 16.


$$\begin{array}{l} \bar{H}=\frac{1}{T}\overset{T}{\underset{t=1}{\sum }}H_{out}\left[t\right] \end{array}$$ (16)

The entire GSR feature extraction process can be summarized as:


$$\begin{array}{l} Z_{GSR}=Transformer\left(Conv1D\left(X_{GSR}^{\prime }\right)\right) \end{array}$$ (17)

Eye movement signals exhibit highly complex temporal characteristics, encompassing both static fixation-related information and dynamic saccadic and microsaccadic behaviors. The signals can reveal subtle changes in cognitive processing and emotional responses. The paper introduces a pure Transformer-based architecture that integrates global attention mechanisms and nonlinear feed-forward networks to more effectively model temporal dependencies and multi-scale dynamic variations in eye movement signals. The overall eye movement feature extraction process can be expressed as:


$$\begin{array}{l} Z_{EYE}=Transformer\left(X_{EYE}^{\prime }\right) \end{array}$$ (18)

After temporal modeling, the aligned GSR and eye movement features are concatenated. The combined features are then pooled to obtain a global representation, which is finally fed into a Softmax classifier to predict the emotional label:


$$\hat{y}=Softmax(FC(GlobalAvgPool(Z)))$$ (19)

## 5 Experiment and result

### 5.1 Experiment settings

The paper employs both the VREED dataset and the self-constructed dataset. VREED is a publicly available multimodal emotion recognition dataset designed for researching emotional modeling in virtual reality environments. It utilizes immersive 360° videos to elicit authentic emotional responses from participants while collecting multimodal data, including eye-tracking data, electrodermal activity, and self-reported emotion ratings. The data collection involved 34 healthy volunteers, each watching videos lasting 1-3 minutes. Emotional states were measured along the dimensions of arousal and valence.

To investigate the performance of the MMTED model under varying task complexities, the study defines two emotion recognition scenarios: a four-class task and an eight-class task.

The Four-class task uses the emotion labels provided by the VREED dataset, emotions were categorized based on arousal and valence levels. All samples were classified into four basic emotional states: high arousal-positive valence, high arousal-negative valence, low arousal-negative valence, and low arousal-positive valence. The classification method is structurally simple and clearly distributed, facilitating the validation of the proposed emotion recognition model's fundamental performance on the public dataset.

The Eight-class tasks uses the self-constructed dataset. A more fine-grained PAD (Pleasure-Arousal-Dominance) emotional space model was adopted, introducing dominance as a third dimension to more comprehensively capture an individual's sense of control and emotional expression in VR contexts.

Based on the tasks, three independent experiments were conducted:

- A four-class task on the VREED dataset to validate the effectiveness of the proposed model;
- An eight-class task on the self-constructed dataset to further verify the MMTED model's efficacy in higher-precision classification;
- A merged four-class task using both VREED and the self-constructed dataset to assess the usability of the self-constructed dataset.

A stratified random splitting strategy was applied to divide the dataset into training (80%) and testing (20%) sets, ensuring consistent distribution of emotional categories across subsets. To achieve efficient emotion classification and evaluation, the following three metrics were selected:

- Accuracy: Measures the proportion of correctly classified samples overall.
- F1-Score: Balances precision and recall, making it suitable for imbalanced class distributions.
- AUC (Area Under the ROC Curve): Evaluates the model's overall discriminative ability across different thresholds, ideal for probabilistic multi-class output tasks.

### 5.2 Results

To comprehensively evaluate the performance of the MMTED model, the study compared it with multiple baseline models, including both traditional machine learning methods and deep learning approaches, on the emotion classification tasks based on the PAD model. The results are presented in Table 4.

**Table 4:** Performance Comparison in Experiment 1, 2, 3.

	**Dataset**	**task**	**Model**	**Accu**	**F1-score**	**AUC**
Experiment 1	VREED	4-Class	SVM	62.38%	0.6082	0.8904
			KNN	53.51%	0.5183	0.7421
			Random Forest	65.12%	0.6385	0.3157
			LightGBM	77.62%	0.7689	0.3986
			LDA	79.23%	0.7827	0.4023
			MMTED	85.52%	0.8428	0.9183
Experiment 2	Self-constructed	8-Class	SVM	66.35%	0.6528	0.8852
			KNN	58.24%	0.5783	0.8031
			Random Forest	61.87%	0.5924	0.9015
			LightGBM	59.12%	0.5726	0.8756
			LDA	65.83%	0.6392	0.9189
			CNN Transformer (GSR only)	82.15%	0.7521	0.9326
			Transformer (Eye only)	84.76%	0.7812	0.9518
			MMTED	89.27%	0.8421	0.9865
Experiment 3	VREED + Self-constructed	4-Class	SVM	58.67%	0.5726	0.7584
			KNN	51.72%	0.5274	0.7389
			Random Forest	64.39%	0.6391	0.8826
			LightGBM	62.54%	0.6226	0.8613
			LDA	55.59%	0.5538	0.4682
			MMTED	85.29%	0.8432	0.9428

**Figure 5:**
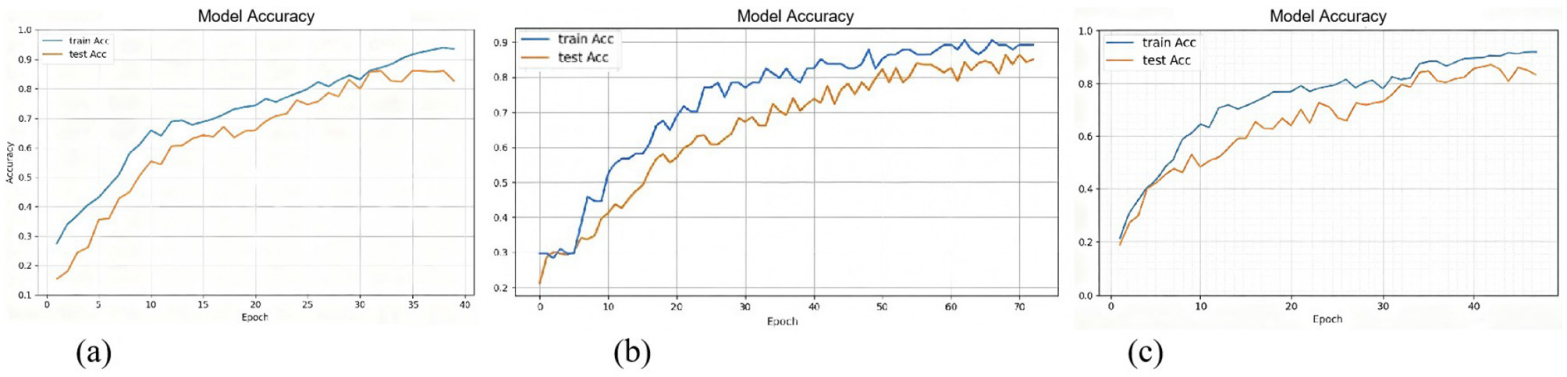
Training accuracy iteration plot for MMTED.

The results of **Experiment 1** demonstrate the MMTED model achieved the highest accuracy of 85.52%, reflecting its strong capability in capturing emotional features through end-to-end modeling. Additionally, it attained an F1-score of 0.8428 and an AUC value of 0.9183, indicating a substantial advantage over traditional machine learning methods. The iterative accuracy curve during model training is illustrated in Figure 5a.

According to the results of **Experiment 2**, MMTED outperformed traditional methods across all three evaluation metrics, demonstrating its stronger modeling capability in handling non-linear, high-dimensional physiological time-series signals. Moreover, as shown in the Table 4, the accuracy achieved through multimodal data fusion (89.27%) was significantly higher than that of single-modality approaches (GSR: 82.15%; Eye: 84.76%). This once again validates the effectiveness of MMTED's multimodal fusion and underscores the strong complementary relationships among different modalities in multimodal emotion recognition tasks. The training accuracy iteration curve of the fusion model is illustrated in Figure 5b.

Furthermore, we observed certain class imbalance issues in the self-constructed dataset (Table 5). To address the issue, the experiment employed Focal Loss in place of cross-entropy loss. While cross-entropy assigns equal weight to every sample, Focal Loss reduces the loss contribution from well-classified samples (those with high confidence) while maintaining higher loss for hard-to-classify examples. For a sample *i* with true class *y*_*i*_ and predicted probability *p*_*t, i*_ for the target class, Focal Loss is defined as follows (Equation 20):


$$\begin{array}{l} FL\left(p_{t,i}\right)=-\alpha_{t}{\left(1-p_{t,i}\right)}^{\gamma }log\left(p_{t,i}\right) \end{array}$$ (20)

where *p*_*t, i*_ denotes the probability that the *i*^*th*^ sample is classified correctly, at is a balancing factor for class *t*, and γ (often set to 2 Lin et al., 2017) is the focusing parameter that controls the emphasis on hard-to-classify samples. When γ = 0, Focal Loss is equivalent to cross-entropy; when γ>0, it reduces the contribution of easy-to-classify samples to the total loss, thereby focusing optimization on hard examples. Adjustments were also made to the optimizer and learning rate during training. The optimization process is detailed in Table 6.

**Table 5:** Number of samples for each of the 8 categories.

**PAD**	**Number of samples**
+P+A+D	59
+P+A−D	54
+P−A+D	36
+P−A−D	28
−P+A+D	22
−P+A−D	42
−P−A+D	65
−P−A−D	60

**Table 6:** Learning rate and loss function optimization.

**Loss**	**LR**	**Accu**	**F1-score**	**AUC**
Focal Loss	0.0001	86.45%	0.8321	0.9753
Focal Loss	0.0003	84.12%	0.8128	0.9632
Focal Loss	0.0005	89.21%	0.8512	0.9824
Cross-entropy	0.0001	88.92%	0.8621	0.9815
Cross-entropy	0.0005	89.27%	0.8421	0.9865
Cross-entropy	0.0007	75.84%	0.7243	0.8921

To validate the model's generalization capability and robustness across different scenarios, the study merged data from the VREED dataset and the self-constructed dataset to form an aggregated dataset. The classification performance of various models was compared on the combined dataset under a four-class emotion recognition task (**Experiment 3**). The experiment compared traditional machine learning methods, single-modality deep learning models, and multimodal fusion models. The results are presented in Table 4, which shows that MMTED achieved an accuracy of 85.29%, significantly outperforming all other models. The training accuracy iteration curve of the fusion model is illustrated in Figure 5c.

When comparing the results across all three experiments, we observed that MMTED achieved the highest accuracy on the self-constructed eight-class task (Acc = 89.27%, F1 = 0.8421, AUC = 0.9865), while its performance was lowest on the mixed-dataset four-class task (Acc = 85.29%, F1 = 0.8432, AUC = 0.9428). The discrepancy can be attributed to differences between the two datasets in terms of acquisition devices, scenario design, participant demographics, and emotion elicitation intensity. The inconsistencies led to a degradation in model performance when generalizing across domains, making it challenging for the fusion model to stably extract cross-modal features from the mixed samples.

For each experiment, we conducted 5-fold cross-validation, where each fold maintains the 80/20 split proportion. The results are shown in Table 7.

**Table 7:** 5-fold cross-validation of experiment 1, 2, 3.

	**Dataset**	**Task**	**Model**	**Accu**	**F1-score**	**AUC**
Experiment 1	VREED	4-Class	SVM	59.38%	0.5713	0.8958
			KNN	62.19%	0.8108	0.7029
			Random Forest	61.90%	0.6206	0.8546
			LightGBM	74.60%	0.7447	0.9190
			LDA	76.19%	0.7636	0.3884
			MMTED	85.94%	0.9125	0.9725
Experiment 2	Self-constructed	8-Class	SVM	67.38%	0.6713	0.7962
			KNN	54.05%	0.5269	0.8226
			Random Forest	55.41%	0.5279	0.8754
			LightGBM	54.05%	0.5145	0.4754
			LDA	70.89%	0.6717	0.8299
			MMTED	90.76%	0.9253	0.9711
Experiment 3	VREED + Self-constructed	4-Class	SVM	55.88%	s 0.5453	0.7392
			KNN	57.53%	0.5754	0.7232
			Random Forest	59.56%	0.5930	0.8420
			LightGBM	63.24%	0.6313	0.8688
			LDA	53.94%	0.5274	0.4429
			MMTED	85.78%	0.8925	0.9520

**Figure 6:**
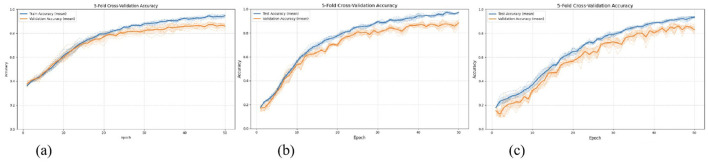
Training accuracy iteration plot for MMTED in 5-fold cross-validation. **(a)** Experiment 1: MMTED, **(b)** Experiment 2: MMTED, **(c)** Experiment 3: MMTED.

The average accuracy across folds was within 1–2% of the 80/20 split result (Table 7), demonstrating stability. And the iterative accuracy curve during model training is illustrated in Figure 6.

The results highlight the complexity and challenges of cross-dataset modeling in physiological signal-based emotion recognition. They also suggest that future research should consider incorporating domain adaptation mechanisms or data standardization methods to enhance the model's generalization capability in multi-source data scenarios.

### 5.3 Ablation studies

Ablation studies were conducted to analyze each module's contribution to the multimodal model's performance, involving systematic removal of attention modules, individual modality channels, and fusion layers (Table 8). To evaluate the multimodal approach, the fusion model was compared against two unimodal baselines: a CNN-Transformer for electrodermal activity and a Transformer for eye-tracking data. The multimodal architecture integrates deep semantic features from both modalities while employing attention mechanisms to dynamically weight features, thereby enhancing classification performance.

**Table 8:** Ablation study analysis.

**Model**	**Attention**	**Multi**	**Accu**	**F1-score**
MMTED	Yes	Yes	89.27%	0.8421
MMTED (no attenion)	No	Yes	86.46%	0.7824
GSR-only	-	No	82.15%	0.7521
Eye-only	-	No	84.76%	0.7812

**Figure 7:**
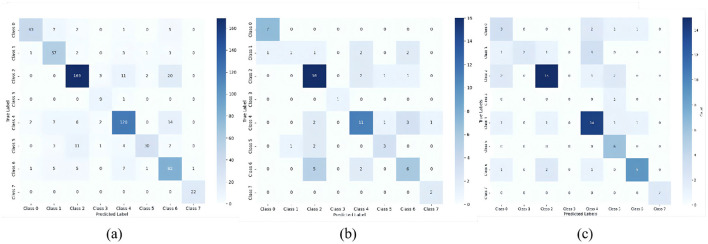
Confusion matrices of MMTED model vs. GSR-only models vs. eye-only models. **(a)** MMTED Model, **(b)** GSR-Only Models, **(c)** Eye-Only Models.

In confusion matrices, the multimodal model exhibits stronger diagonal concentrations (Figure 7), indicating more confident predictions and well-defined decision boundaries across most emotion classes. Conversely, the eye-tracking unimodal model shows substantial off-diagonal activation, revealing higher classification uncertainty and vulnerability to sample variations or feature deficiencies. This demonstrates the fusion strategy's effectiveness in mitigating overfitting/underfitting limitations of unimodal approaches while improving generalizability.

Experimental results confirm the attention mechanism's significant impact, boosting accuracy by 3.6% and validating its feature-weighting efficacy. Furthermore, single-modality ablation tests prove bimodal synergy substantially outperforms either isolated modality, with the fusion layer playing a critical role in integrating heterogeneous features.

Collectively, the proposed fusion model not only surpasses baselines in overall performance but also validates the contribution of each component (multimodal integration, deep feature extraction) to its superiority, establishing a transferable framework for future multimodal affective computing research.

## 6 Conclusion

The shortage of datasets and the limited generalizability of multimodal models have posed significant challenges to emotion research in VR. To address the lack of VR emotion recognition datasets, the study expands the VREED dataset through new experiments. We developed VR scenarios for emotion induction, integrating multimodal stimuli such as auditory and visual content to elicit users' emotional responses across different dimensions. A total of 38 participants were recruited and required to watch 10 carefully selected videos, during which their electrodermal activity (EDA) and eye-tracking data were continuously recorded. Based on the eye-tracking signals, dynamic metrics such as fixations, saccades, and blinks were extracted. The extracted features encompass time-domain statistics, frequency-domain measures, and dynamic behavioral indicators, collectively forming the input vectors required for model training.

To improve the accuracy of emotion recognition, the paper designed a novel multimodal emotion recognition approach named MMTED, which integrates convolutional neural networks (CNN), Transformer, and attention mechanisms. By combining subjective dimensions of emotion modeling with objective signal features, a more discriminative recognition framework was constructed. During model training and analysis, single-modality and multi-modality strategies were compared, validating that multi-source signal fusion enhances emotion recognition performance. An end-to-end modeling approach based on CNN-Transformer and pure Transformer architectures was applied to process multimodal signals directly from raw physiological data to emotional states. Experimental results showed that MMTED achieved a highest average accuracy of 89.27% in multi-class tasks, outperforming traditional machine learning models. Ablation studies analyzing the contribution of attention mechanisms and each modality further revealed the effectiveness and complementarity of multimodal fusion. In response to the need for personalized feedback in VR scenarios, an adaptive emotional feedback mechanism was proposed, providing a theoretical foundation for future personalized emotional interaction in VR environments.

While the study has achieved certain results in physiological signal-based emotion recognition in VR settings, several limitations and areas for improvement remain. The sample size and data diversity were limited, and issues such as data distribution bias, data augmentation, and model generalization capability need further addressing.

## Data Availability

The raw data supporting the conclusions of this article will be made available by the authors, without undue reservation.

## Footnotes

1
https://dl.acm.org/doi/10.1145/3495002

2
https://github.com/JuneQCX/Emotion-Inducing-Video-Library
